# Data-Driven Crystal Structure Prediction for Ternary Metal Chalcogenides

**DOI:** 10.1021/acs.chemmater.5c02077

**Published:** 2025-12-22

**Authors:** Tianshu Li, Hyunsoo Park, Aron Walsh

**Affiliations:** Department of Materials, 4615 Imperial College London, Exhibition Road, London SW7 2AZ, U.K.

## Abstract

The efficient design and discovery of stable inorganic crystal structures is central to materials innovation. Here, we compare data-driven approaches for accelerated crystal structure prediction: substitution into known prototype structures, generative artificial intelligence (genAI) using denoising diffusion as implemented in Chemeleon, and an evolutionary global optimization search. Candidate structures are optimized using an ensemble of machine-learned interatomic potentials, providing both energy estimates and uncertainty quantification. Applied to a large set of known and hypothetical ternary metal chalcogenide compositions, including technologically relevant sulfides such as Na_2_SiS_3_, RbPS_3_, and KMo_2_S_4_, our analysis reveals that the genAI approach not only matches but can surpass traditional methods in identifying diverse, low-energy structures. These findings highlight the promise of generative models for scalable structural exploration of inorganic materials space.

## Graphical abstract

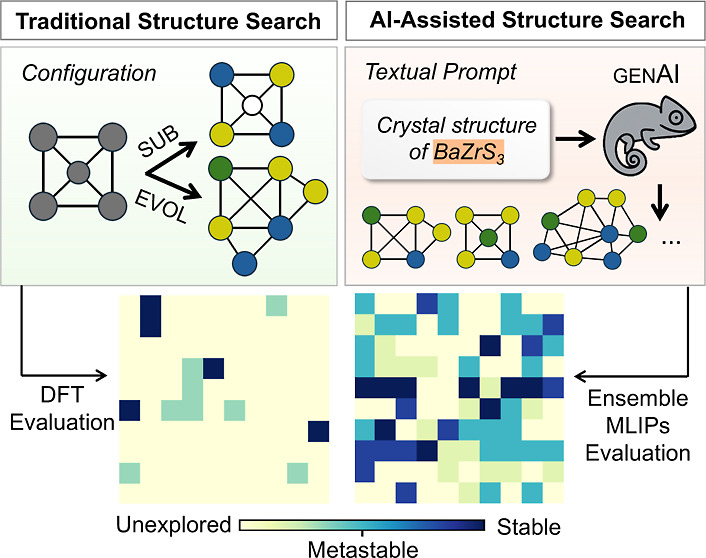

## Introduction

Predicting stable crystal structures directly from chemical compositions has been a longstanding objective in materials science.^1^ Traditional computational efforts toward this goal have primarily relied on physics-based global optimization techniques, including evolutionary algorithms (e.g., USPEX^2,3^ ), random structure searching (e.g., AIRSS^4,5^ ), and particle swarm optimization (e.g., CALYPSO^6,7^ ). Such methods explore the configuration space by generating trial structures and optimizing them based on total energy evaluations, typically guided by symmetry heuristics or physical constraints such as bond lengths and coordination environments. Notably, they have succeeded in uncovering structures that emerge under extreme or nonequilibrium conditions, such as high-T_C_ superconductors,^8–10^ superhard polymorphs,^11,12^ and kinetically stabilized phases.^13,14^ Nevertheless, these approaches remain computationally demanding due to the vastness of the structural search space, the poor scaling with increasing complexity, and the reliance on high-throughput first-principles calculations for energy evaluations.

The growth of crystal structure databases such as the Materials Project (MP),^15^ Inorganic Crystal Structure Database (ICSD),^16^ and NOMAD^17^ has enabled data-mining strategies to emerge as viable alternatives to traditional structure prediction methods. A widely adopted approach is prototype-based structure substitution, where known stable or metastable structures serve as templates for generating novel compounds through systematic elemental substitution across the periodic table.^18^ This strategy has been remarkably successful in accelerating the discovery of functional materials, including potential candidates for photovoltaics,^19,20^ solar fuels,^21^ thermoelectrics,^22–24^ and Li-ion battery cathodes.^25^ While this method drastically reduces the search space and is well-suited for high-throughput screening, it remains inherently constrained by the structural diversity of the prototype pool and is less effective at discovering compounds with novel crystal topologies.^26^

More recently, generative artificial intelligence offers an alternative approach for data-driven crystal structure prediction and materials design. Prior work has shown that locally stable atomic configurations tend to lie on low-dimensional manifolds embedded within the high-dimensional configuration space.^27^ Leveraging this insight, various generative architectures, including Variational Autoencoders (VAEs), Generative Adversarial Networks (GANs), denoising diffusion models, and autoregressive Transformers, have been developed to sample chemically plausible structures by learning the complex distributions from curated crystal structure databases.^26^ These models differ in how they encode, sample, and decode structures, offering complementary computational strategies. For example, Crystal Diffusion Variational Autoencoder integrates a generative denoising diffusion model as the decoder within the VAE architecture, and demonstrated valid, diverse, and physically plausible structure generation under both ambient and high-pressure conditions.^28–30^ The application range of generative structure models has been expanding, including amorphous solids,^31^ ternary alloys,^32,33^ and superconductors.^34^

Building on recent advances in structure prediction techniques, we direct our attention to the ternary metal chalcogenides, a class of compounds with promise in thermoelectrics, optoelectronics, topological phases, and electronics.^35–39^ Compared to metal oxides, chalcogenides feature stronger covalent interactions between metal and chalcogen atoms, leading to greater orbital overlap and more delocalized valence bands. These characteristics contribute to electrical conductivity through lower hole effective masses and enhanced carrier mobility.^40^ For instance, copper-based chalcogenides represented by NaCu_4_Se_4_, have demonstrated a carrier density of ∼10^21^ cm^–3^ and a high hole mobility up to 808 cm^2^ V^–1^ s^–1^, significantly outperforming the majority of Cu-based oxides, which typically exhibit mobilities below 1 cm^2^ V^–1^ s^–1^.^41^ Additionally, the elevated energy of chalcogen-p orbitals raises the valence band maximum, facilitating p-type doping and further improving their suitability for optoelectronic and thermoelectric applications. However, despite these favorable electronic properties, the experimental exploration of multicomponent metal chalcogenides has lagged behind that of oxide counterparts, partly due to synthetic challenges with strong phase competition.

In this work, we evaluate the performance of the denoising diffusion-based generative model Chemeleon^42^ against prototype-based structure substitution and global evolutionary search methods. Our evaluation focuses on both the structural diversity and thermodynamic stability of predicted phases across a set of ternary metal chalcogenide compositions of varying chemical complexity. To ensure reliable relaxation to local minimum structures, we employ an ensemble of machine learning force fields that provide energy estimates with uncertainty quantification. Our results show that generative techniques are not only competitive with traditional approaches but can outperform them in sampling structurally diverse, low-energy configurations. Underpinning the generation of low energy structures is the preferential sampling of tetrahedral and octahedral coordination environments for the metal cations, matching expectations from materials chemistry, and the distribution of known atomic environments.

## Methods

### Crystal Structure Generation

#### Prototype Substitution (sub)

Candidate compounds are produced by replacing atomic species within known crystal structure prototypes. Hautier et al. proposed an ionic substitution algorithm,^18,43^ which has proven effective for accelerating material exploration and discovery over the past decade.^44,45^ Drawing from the MP database (version 2024.12.18), a collection of 30 prototype structures and a series of stable ternary sulfides generated via the sub method detailed in our previous work^19^ were selected as templates for ternary chalcogenide exploration.

#### Generative Artificial Intelligence (genAI)

Structure generation was conducted using Chemeleon,^42^ a text-guided denoising diffusion framework trained based on stable crystal structures sourced from MP. This artificial intelligence approach allows atomic coordinates and lattice parameters to be sampled directly from composition as text representation, without relying on predefined structural prototypes. For each composition A_*x*_B_*y*_S_*z*_, Chemeleon generated candidate structures across multiple stoichiometries, with a maximum total atom count of up to 40 atoms per unit cell. For each stoichiometry, we sampled 100 candidate structures. To eliminate redundancy, duplicate structures were filtered using the StructureMatcher function of Pymatgen^46^ with the following matching criteria: a fractional length tolerance of 0.2, a site tolerance of 0.3 Å, and an angle tolerance of 10°. Structures satisfying these criteria were considered as duplicates and removed from further analysis.

#### Evolutionary Structure Search (Evol)

To provide an additional point of comparison, we performed an evolutionary search using MAGUS (Machine learning And Graph theory assisted Universal structure Searcher), which applies global optimization through genetic operations such as heredity, permutation, and mutation, guided by energy-based fitness.^47,48^ An evol search with a machine learning interatomic potential (MLIP) used as the energy calculator was applied to KBiSe_2_ with unit cell multiplication factors ranging from 1 to 10. For each stoichiometry, 20 generations were performed, with each generation sampling 50 candidate structures.

### Structure Analysis

To evaluate the structural validity of the generated structures, we adopted two complementary approaches. Local coordination environments were characterized by site fingerprints computed through CrystalNN function in Pymatgen^46^ and matminer.^49^ These site-level features were aggregated into structure-level fingerprints, and pairwise Euclidean distances with known lowest-energy phases in the MP database were computed to quantify the structural dissimilarity.^50^ For structural diversity and clustering, Smooth Overlap of Atomic Positions (SOAP) descriptors^51,52^ were calculated for each structure and embedded into a two-dimensional space using the t-distributed stochastic neighbor embedding (*t*-SNE) projection.

### Geometry Optimization and Thermodynamic Stability

To enable efficient and accurate evaluation of structural energetics, we utilized MLIPs for geometry optimization and thermodynamic stability analysis. To guide the geometry relaxation along a physically meaningful path, we employed an ensemble of three different machine learning interatomic potentials, used iteratively: MACE-MPA-0,^53,54^ MatterSim,^55^ and GRACE-2L-OAM.^56^ We incorporated the standard deviation of the predicted forces (α_F_) as an uncertainty estimate to prevent the structure from deviating across different potentials, as illustrated in eqs 1 and 2.

For a given configuration, the mean force magnitude predicted by the *k*-th model is

1 $$F_{k}=\frac{1}{M}\sum_{i=1}^{M}∥F_{i}^{\left(k\right)}∥=\frac{1}{M}\sum_{i=1}^{M}\sqrt{{\left(F_{i,x}^{\left(k\right)}\right)}^{2}+{\left(F_{i,y}^{\left(k\right)}\right)}^{2}+{\left(F_{i,z}^{\left(k\right)}\right)}^{2}}$$

where *M* is the number of atoms and **F**_*i*_^(k)^ is the force vector on atom *i* predicted by model *k*. The corresponding uncertainty estimate across the *N* models in the ensemble is given by

2 $$\mathrm{F̅}=\frac{1}{N}\sum_{k=1}^{N}F_{k},\;\alpha_{F}=\sqrt{\frac{1}{N}\sum_{k=1}^{N}{\left(F_{k}-\mathrm{F̅}\right)}^{2}}$$

where *N* is the number of MLIPs in the ensemble and *F̅* is the mean force magnitude over models. At each optimization step, atomic forces were predicted using the three models. The average force magnitude *F*_*k*_ was computed for each model, and the model with the lowest *F*_*k*_ was selected as the calculator for that step. Simultaneously, the force field used for relaxation was refined by averaging the per-atom force vectors across all models, prioritising atoms with high local stress while maintaining physically reasonable positions.

Geometry relaxation was carried out using the Fast Inertial Relaxation Engine algorithm,^57^ with the convergence determined when the maximum force acting on any atom does not exceed 0.01 eV/Å. Cell and internal relaxations were performed iteratively for structures with degrees of freedom. In addition to the force-based criterion, we also considered the energy-based criterion in the ensemble and selected the final relaxed structure with the lowest predicted energy. This dual consideration of force norm and energy ensures both stable convergence and thermodynamic consistency in the optimization. The comparisons among ensembled MLIPs, single MLIP using GRACE-2L-OAM model, and calculations based on density functional theory (DFT) are shown in Figure S1a,b.

We applied GRACE-2L-OAM to predict the final energy, including the formation energy (*E*_*f*_) and the energy above the convex hull (*E*_hull_). As shown in Figure S1c, among three interatomic potentials involved, GRACE-2L-OAM achieves the lowest mean absolute error (MAE) in predictions relative to DFT. To ensure consistency with the MP database, the GRACE-2L-OAM model was used to recompute the energies of both the target structures and their corresponding known competing phases. This can reduce energy discrepancies arising from different computational methods and parameters, ensuring a consistent stability assessment. The details of DFT calculations are provided in the Supporting Information.

## Results and Discussion

We begin by evaluating the performance of the generative and substitution approaches. We perform a comparison across three test sets: (1) 30 known ternary metal chalcogenide compositions whose ground-state structures correspond to the most frequently occurring prototypes in the MP database; (2) 416 ternary metal sulfide compositions previously generated with at least one stable phase using the sub strategy;^19^ and (3) the full set of 3,286 unique ternary metal chalcogenide compositions from the MP database, each with a conventional unit cell containing fewer than 40 atoms.

### Known Test Set

We start our evaluation using 30 ternary chalcogenide compositions with known common structures. The lowest-energy structures for these compositions serve as benchmarks to assess each method’s ability to generate geometrically diverse structures. Each structure generated by Chemeleon is represented by a circle in Figure 1a, with the *y*-axis denoting its structural dissimilarity to the corresponding MP prototype. Values of dissimilarity approaching zero reflect minimal structural differences, whereas ones above 1.0 imply substantial geometric deviation.^50^ The color of each point encodes the predicted *E*_hull_. For comparison, red triangles indicate stable structures obtained via the sub method. It is worth noting that the sub-derived stable structures are not always generated from the same structural prototypes as the lowest-energy phases reported in the MP database, since compounds with identical composition may originate from distinct prototype frameworks. Substituted structures derived from alternative prototypes can become stable after geometry relaxation, resulting in noticeable structural dissimilarities from the MP reference. While substitution confines generation to the vicinity of known prototypes thus avoiding high energy configurations, genAI operates without such constraints, allowing structures to be sampled from learned structural features. genAI explores a broader range of structural motifs while still recovering low-energy structures that resemble known prototypes. To further investigate the diversity of atomic environments, we compute SOAP descriptors for each structure and visualize the results using a *t*-SNE projection, as shown in Figure 1b. Each point represents a structure mapped from high-dimensional SOAP features onto a 2*D* plane. The distribution reveals that Chemeleon-generated structures not only recover atomic environments found in MP and substitution-derived structures, but also populate unexplored regions of configuration space. This indicates the capacity to balance structural fidelity with creativity in crystal structure generation, confirming that novel polymorphic structures originate from generative behavior rather than direct recall from the training set. More information, including MP IDs and the genAI sampling distribution for each composition, is provided in Figures S2 and S3.

**Figure 1:**
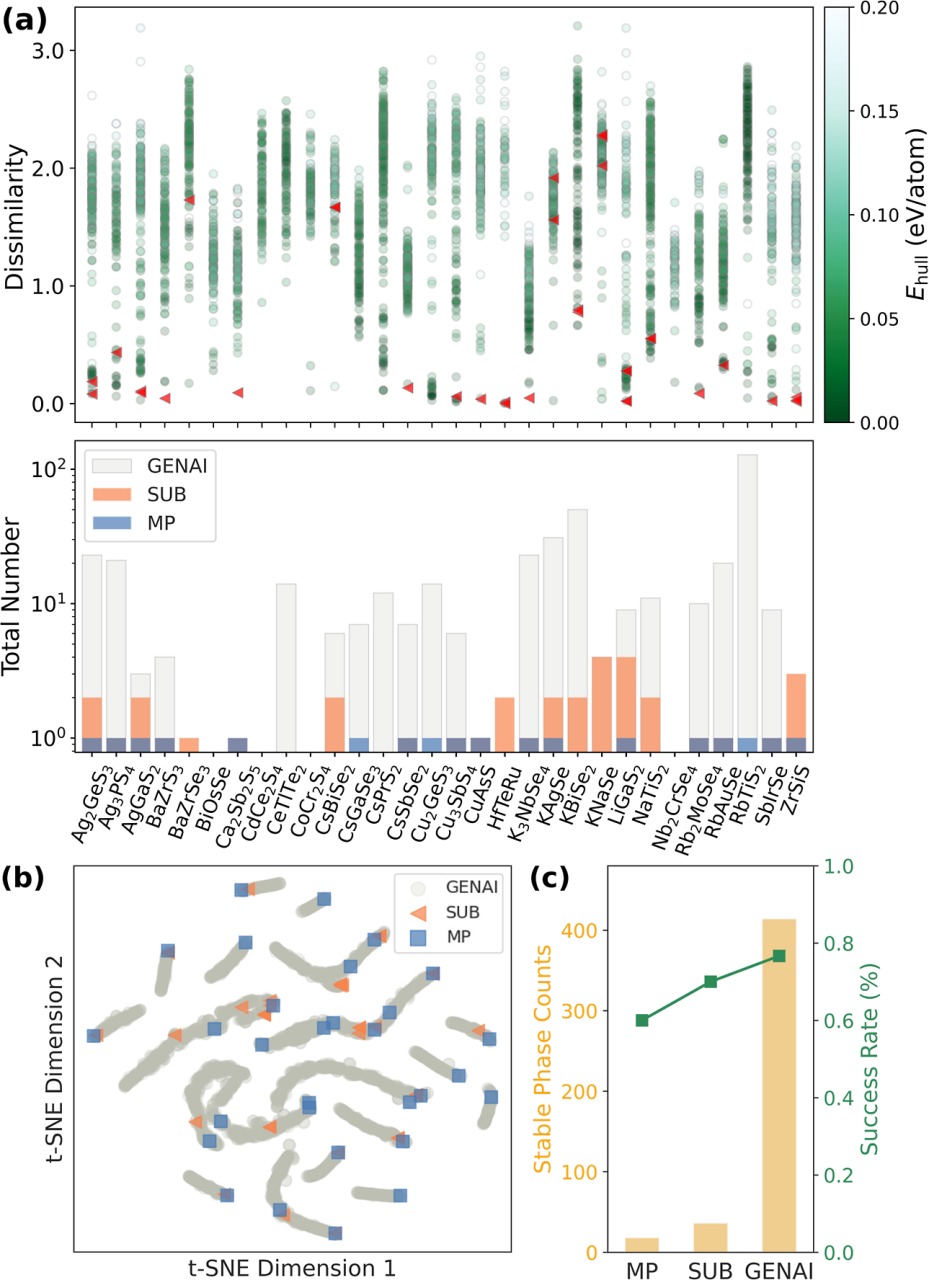
Comparative evaluation of structural diversity and stability across 30 ternary chalcogenide compositions. (a) (Upper panel) Structural similarity between each generated structure and its corresponding lowest-energy structure from the MP database, colored by *E*_hull_. Red triangles mark the stable phases generated by sub; (lower panel) Statistics of thermodynamically stable structures (*E*_hull_ < 0.02 eV/atom) identified per composition across three data sets. (b) *t*-SNE embedding of SOAP descriptors illustrating the structural distribution of genAI (gray circles), sub (orange triangles), and MP-recorded lowest-energy structures (blue squares). (c) Comparison of the total number of stable phases (left axis) and the success rate in identifying stable phases (right axis) across three data resources.

To evaluate the ability of Chemeleon to generate thermodynamically stable phases, we quantified the number of stable structures identified by each method. To account for intrinsic prediction errors of MLIP, we adopt two criteria for thermodynamic stability evaluation:(1) *E*_*f*_ < 0, and (2) *E*_hull_ < 0.02 eV/atom. This threshold is chosen to be within the MAE of the GRACE-2L-OAM model to predict convex hull energies, ensuring that the structures identified as stable are not affected by model uncertainty.^58^ This energy threshold is consistently applied across all stable phase identification processes using the same model to ensure uniformity in stability screening. Among the 30 target compositions, 18 exhibit known stable phases recorded in the MP database (60% success rate in Figure 1c). The sub method yields stable structures for 21 compositions, resulting in a total of 36 unique low-energy configurations in the chemical space (70% success rate). While this approach improves coverage relative to MP alone, the number of stable phases remains modest.

In contrast, the generative model produces stable structures for 23 compositions, increasing the overall success rate to 77%, and generating 414 distinct configurations with *E*_hull_ values below the threshold. The *E*_hull_ distributions of the generated structures are compared between the sub (blue) and genAI (orange) approaches, as shown in Figure S4. sub tends to sample near existing local minima on the potential energy surface, efficiently yielding low-energy structures. Interestingly, our results indicate that the genAI approach exhibits a comparable capability in identifying low-energy structures, while simultaneously exploring a broader configurational space. To evaluate whether increasing the prototype pool can enhance the performance of the sub approach, we further expand the prototype library based on the statistical distribution and structural dissimilarity in the MP database. For each selected prototype, elemental substitutions were carried out according to the target stoichiometry, and when multiple elements shared the same ratio, all possible substitution combinations were considered. The chalcogen atoms in each prototype were replaced by the corresponding chalcogen species in the target composition. Each substituted structure was then optimized and its thermodynamic stability was evaluated using MLIPs, following the same workflow as that used for genAI process. As shown in Figure S5, the number of compositions yielding stable phases increased from 5 to 21 as the prototype pool expanded from 7 to 30. Further enlarging the pool to 40 and beyond produced only two additional stable phases (RbTiS_2_ and CeTlTe_2_), both of which were also generated as stable structures by genAI. These results indicate that the initial set of 30 prototypes exhibits a sufficient structural diversity for the sub, while further expansion yields limited gains. The relative performance of genAI underscores the model’s ability to more thoroughly explore the potential energy landscape and identify a broad spectrum of low-energy local minima that prototype-guided methods may overlook.

### Substitution Test Set

We next evaluate the performance on a broader benchmark set of 416 ternary sulfide compositions (A_*x*_B_*y*_S_*z*_), for which the sub method successfully identified at least one stable phase in our previous work.^19^ Our objective is not only to verify whether the generative model can recover known stable phases, but also to assess its potential to discover alternative configurations with lower formation energies.

To ensure consistency and eliminate discrepancies between first-principles and surrogate models, we recalculated the thermodynamic stability of all substitution-derived stable structures using GRACE. After filtering, 376 compositions retained at least one stable phase with *E*_hull_ below the defined energy threshold. For compositions, we applied Chemeleon to sample structures for this reduced set of chemical compositions. The model successfully identified at least one stable configuration for 354 out of the 376 compositions, corresponding to a high success rate of 94%, and yielding a total of 814 unique low-energy structures. For 203 of these compositions, Chemeleon generated structures with lower predicted *E*_*f*_ than those obtained via sub method, indicating a systematic ability to access deeper minima on the potential energy surface. In contrast, the substitution approach yielded lower-energy structures in only 92 cases, underscoring the enhanced discovery potential of the generative framework.

To further examine the energetic advantage conferred by genAI, we visualize the formation energy differences between the lowest-energy structures using a heatmap grouped by A- and B-site elements, as shown in Figure 2. Negative values (in blue) indicate compositions where genAI discovered structures with lower *E*_*f*_, while positive values (in red) mark cases where sub approach yielded lower-energy configurations. The intensity of the color reflects the magnitude of the *E*_*f*_ difference between the lowest-energy structures obtained by genAI and sub for the same composition. Blank regions indicate that no stable phases were found in this chemical system using our prior sub approach. The identified stable structures of five representative compositions, including RbPS_3_, Na_2_SiS_3_, Ca_2_GeS_4_, AgNaS, and KMo_2_S_4_ are shown on the Figure 2, demonstrating the strong capability of Chemeleon in generating novel structures with reasonable configurations. Notably, the generative model shows a pronounced advantage for compositions containing alkaline-earth elements and early transition metals (e.g., Group 3 and Group 4 species). These elements are known to introduce substantial structural and chemical complexity due to their intrinsic properties.^59,60^ Early transition metals often exhibit variable oxidation states and adopt diverse coordination environments, while the relatively large ionic radii and high electropositivity of alkaline-earth metals promote the formation of flexible crystal frameworks. This flexibility results in a richer set of energetically competitive structures, making these systems particularly challenging for template-driven approaches.

**Figure 2:**
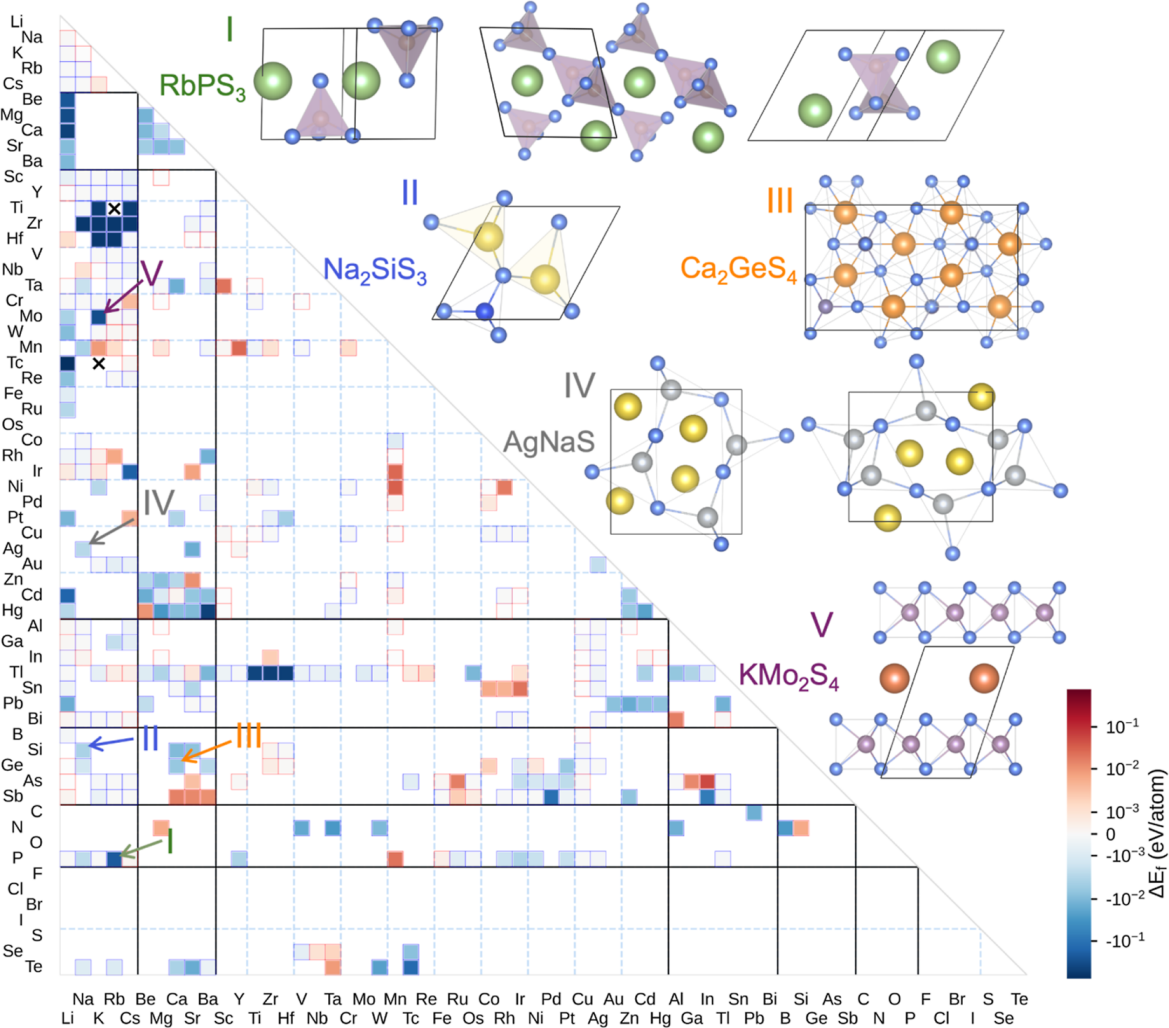
Formation energy difference (Δ*E*_*f*_) between the lowest-energy structures identified by genAI and sub across 416 ternary sulfide compositions. The color intensity corresponds to the magnitude of energy difference (in eV/atom). Negative values (blue corresponds to a lower energy genAI structure). Elements along the *x*- and *y*-axes represent the A-site and B-site species in each ternary composition, respectively. Blank regions correspond to chemical systems without generative sampling performed, whereas crosses denote cases where no structures were generated via genAI. Stable structures generated by genAI for five representative compositions are shown in the upper right.

The prototype-based substitution method is inherently limited to exploring the local vicinity of known structural motifs and thus may fail to access the true global minima on the potential energy surface. In contrast, the generative model is not constrained by predefined prototypes and can sample a broader range of atomic configurations. Moreover, the energy landscapes of transition-metal chalcogenides are often characterized by multiple local minima separated by kinetic barriers. While substitution methods tend to remain trapped near the structural templates, genAI exhibits the capacity to navigate beyond these barriers and propose globally more favorable structures. It is important to note, however, that the stability predictions presented here are based on MLIP that do not explicitly account for spin polarization, which is particularly relevant for systems containing open-shell transition metals. As such, further high-accuracy first-principles evaluations will be necessary to fully validate the stability of potentially magnetic candidate structures.

### Extended Test Set

We next extend our evaluation to a broader data set comprising 3,286 unique ternary chalcogenide compositions of the form A_*x*_B_*y*_Ch_*z*_ (where Ch = S, Se, Te), as compiled from the MP database. Among these, 1,455 compositions are documented to possess at least one thermodynamically stable phase on the convex hull. Using MLIPs for geometry optimization and energetic evaluation, Chemeleon predicted stable structures for 1,665 compositions, 1,093 of which match known stable entries in MP, as shown in Figure 3. The color indicates the *E*_hull_ within the defined energy threshold. This demonstrates strong recall of existing materials, while also revealing the model’s ability to propose novel stable candidates. We further performed structure matching between the generated stable phases (predicted *E*_hull_ = 0 eV/atom) and the known entries in the MP database using the StructureMatcher function. Among the 1,455 compositions, we found that the stable phases of 873 compositions were successfully reproduced by Chemeleon. Remarkably, 51% of the generated stable phases involve transition-metal-containing compositions, amounting to 921 distinct systems. This reflects the model’s capacity to handle chemically and structurally complex material spaces, which are often underrepresented or difficult to explore using conventional approaches. In total, Chemeleon generated 7,517 unique low-energy structures within this composition set, including 6,749 stable candidates (*E*_hull_ = 0 eV/atom) across 1,079 compositions.

**Figure 3:**
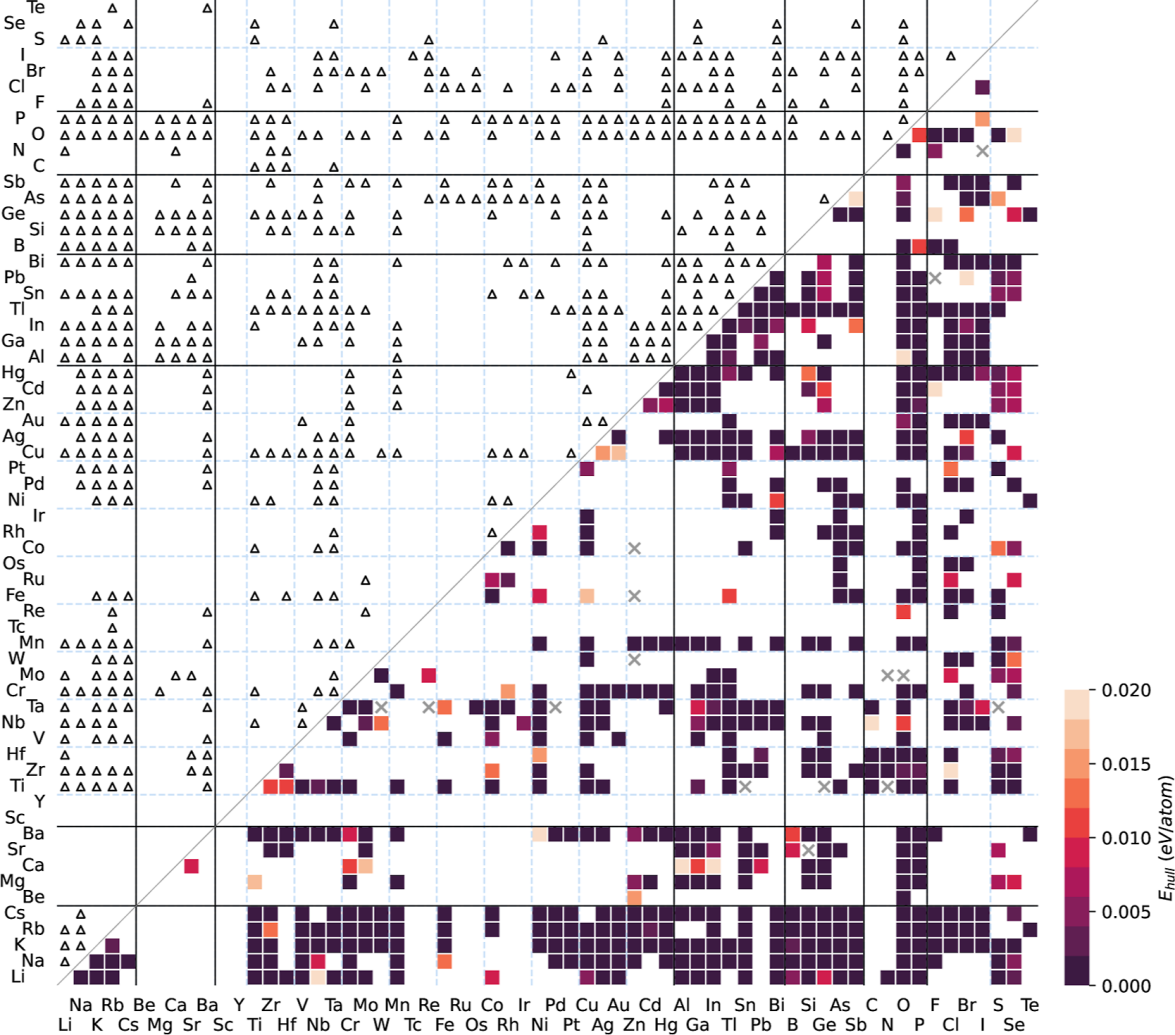
Thermodynamic stability map of the lowest-energy structures generated across 3,286 ternary chalcogenide compositions. Colors indicate the predicted *E*_hull_ (eV/atom), with darker shades representing higher thermodynamic stability. Black triangles mark compositions with confirmed stable phases in the MP database. Blank regions correspond to chemical systems for which no generative sampling was performed, whereas gray crosses denote cases where no generated structures within the corresponding system yield low energy satisfying the stability criterion.

Beyond sampling structures for specific single compositions, genAI also shows promise in reconstructing phase diagrams and identifying thermodynamically favorable regions. In the Ca–Sr–S system, SrCaS_2_ and Sr_3_CaS_4_ are reported as metastable phases in the MP, with *E*_hull_ = 30 and 14 meV/atom, respectively. As illustrated in Figure 4a, genAI uncovers novel stable Sr_3_CaS_4_ and SrCaS_2_ phases, which define key phase boundaries in the compositional landscape (see Figure S6). For each composition, the lowest-energy stable structure and its corresponding phonon spectrum are shown in Figure 4b. No imaginary phonon modes are found, which confirms these structures as true local minima with dynamic stability. However, for this chemical system, previous experimental studies have shown that CaS and SrS tend to form a solid-solution series rather than distinct ordered crystals, as their lattice parameters vary linearly with composition in accordance with Vegard’s law.^61^ This behavior arises from the similar local environments and ionic radii of Ca^2+^ and Sr^2+^, which enable facile substitution within the same lattice. Further investigation of this intriguing system would be valuable, and MLIPs could assist in exploring potential ordering configurations of related phases.

**Figure 4:**
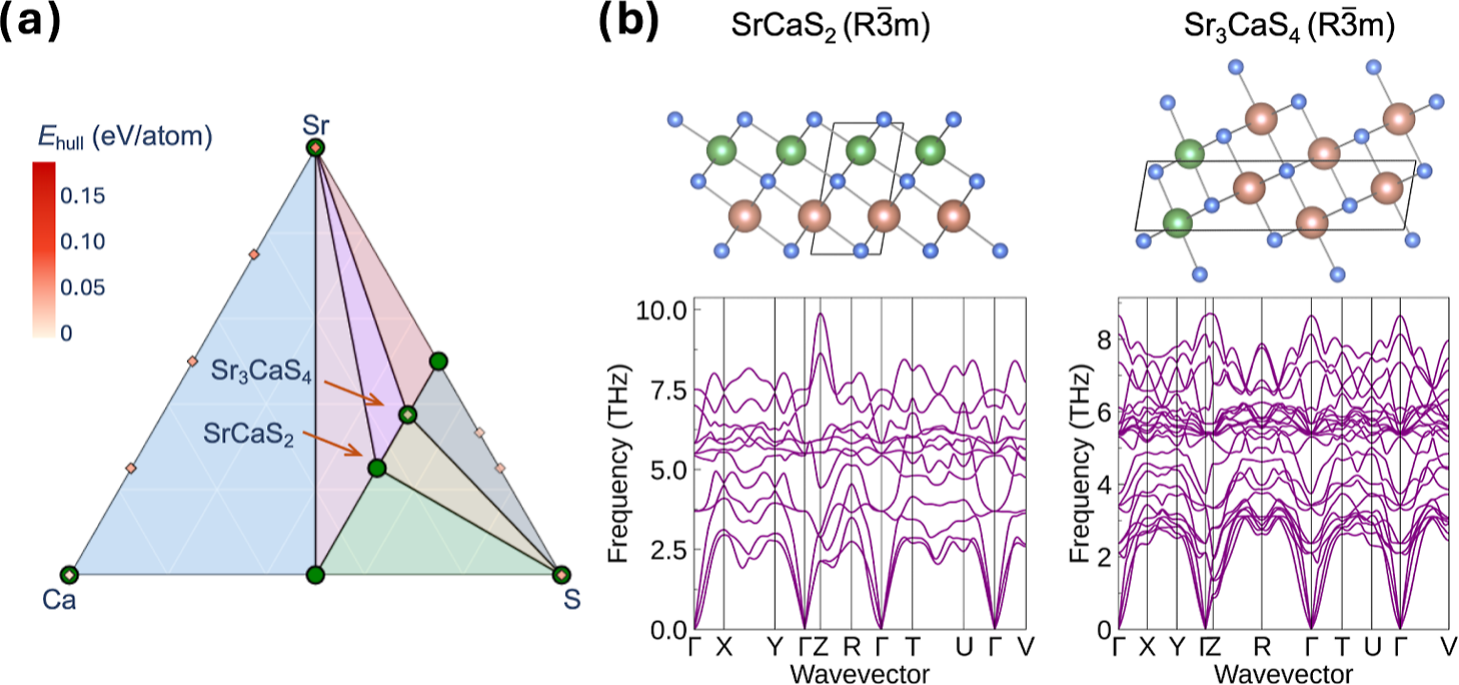
(a) Ternary phase diagrams of the Ca–Sr–S system constructed after incorporating stable structures generated by genAI. Green circles indicate thermodynamically stable phases, while red diamonds represent metastable structures. Color shading reflects the *E*_hull_ in eV/atom, with darker red indicating lower stability. (b) Lowest-energy structures of SrCaS_2_ and Sr_3_CaS_4_, with their corresponding phonon spectra.

While energy is a critical metric for assessing thermodynamic stability, structural validity is also important to ensure that generated configurations are chemically plausible. To this end, we further evaluate the quality of structures produced by genAI in terms of local coordination environments and bond lengths. For the 3,286 target A_*x*_B_*y*_Ch_*z*_ compositions, we treat chalcogen atoms (Ch = S, Se, Te) as anions and examine the coordination numbers of the cations occupying the A and B sites. Atomic coordination numbers were computed using CrystalNN function with a 7 Å distance cutoff for neighbor detection. Our analysis reveals that 91% of stable structures within 3,286 compositions exhibit A- and B-site coordination numbers between 1 and 8, which aligns well with chemically reasonable bonding environments in inorganic solids. Very low coordination numbers (e.g., < 2) typically indicate under-coordinated and unstable atomic arrangements, while unusually high values (>8) are generally associated with *f*-block or large-radius cations in complex oxides or halides, though such cases are rare in our data set. By applying this coordination threshold, we aim to filter out geometrically unrealistic structures and focus on chemically meaningful motifs. As illustrated in Figure 5, for our data set, this version of the Chemeleon model exhibits a bias toward generating structures with triclinic symmetry and local environments dominated by tetrahedral and octahedral coordination. This preference likely reflects the underlying energetic stability of these motifs and their prevalence in stable crystal structures found in the original training data distribution. Given the denoising neural networks in Chemeleon are implemented as equivariant graph neural networks, they effectively capture the local environment around each atom. This capability promotes the generation of chemically reasonable motifs frequently observed in the training data.

**Figure 5:**
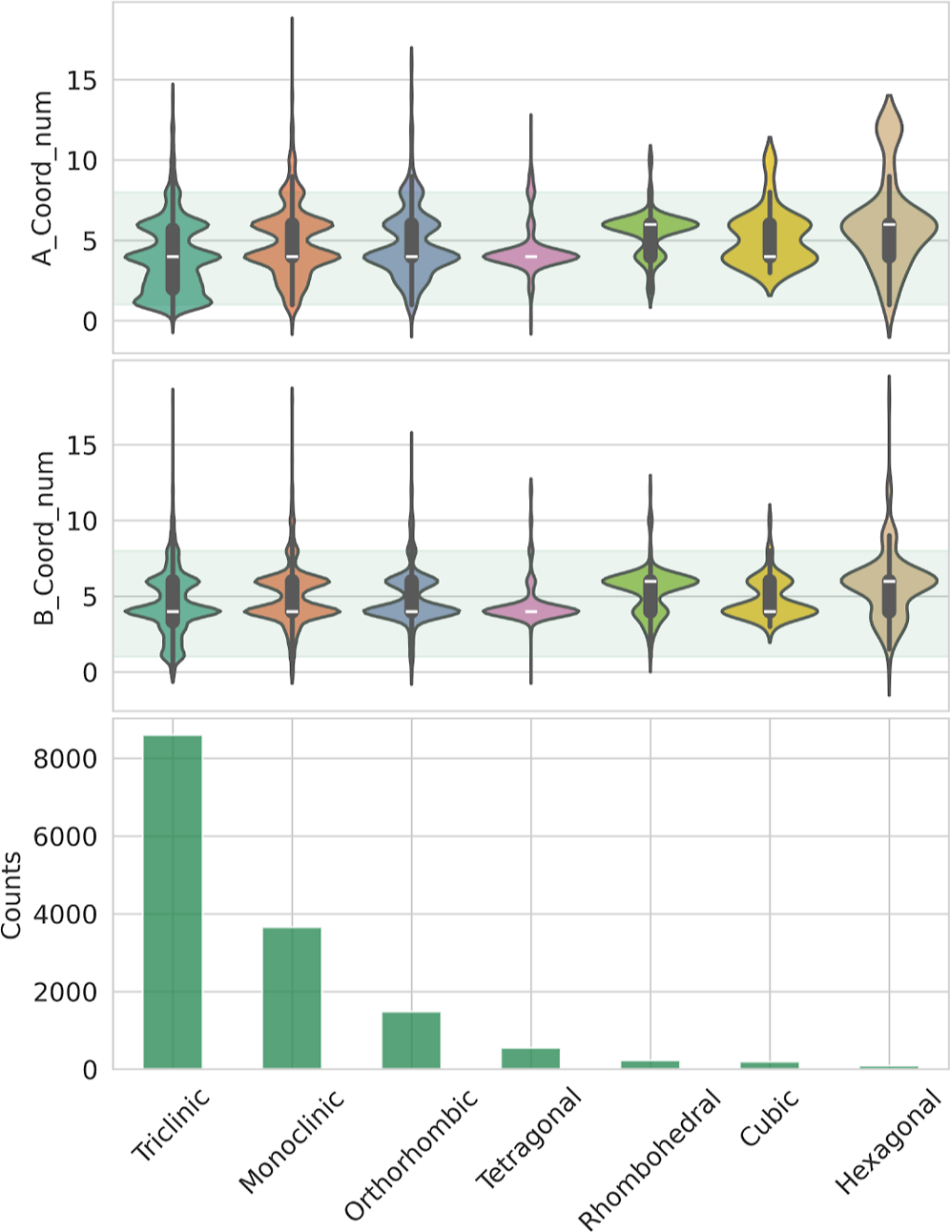
Coordination number and symmetry statistics for genAI-based ternary chalcogenide structures following relaxation. Top and middle panels: Violin plots showing the distribution of coordination numbers for A-site (top) and B-site (middle) cations, grouped by the resulting crystal symmetry. Shaded green regions indicate the chemically common coordination range (1–8). Bottom panel: Histogram of crystal symmetry types, with triclinic and monoclinic systems being the most frequently generated.

### Generation vs Evolution

To further benchmark the structure generation capabilities of the generative model, we compared genAI approach with a typical evol strategy, as implemented in MAGUS package. Both methods were applied to KBiSe_2_, a representative ternary composition that lacks stable phases in the current MP database. We evaluate performance based on two key metrics: (1) the number of unique structures generated across varying unit cell sizes (up to 40 atoms), and (2) the number of thermodynamically stable structures identified, defined by *E*_hull_ below the energy threshold. As illustrated in Figure 6a, genAI and evol display distinct structure generation patterns. In the low-complexity regime (N_atoms_ ≤ 8), both models tend to produce layered configurations built from octahedral KSe_6_ and BiSe_6_ units. These closely resemble known chalcogenide motifs and consistent with the few structures recorded in the MP database for similar compositions (see Figures 6c and S7a,b). This agreement suggests that both methods are guided by effective structural priors when generating compact, symmetric structures.

**Figure 6:**
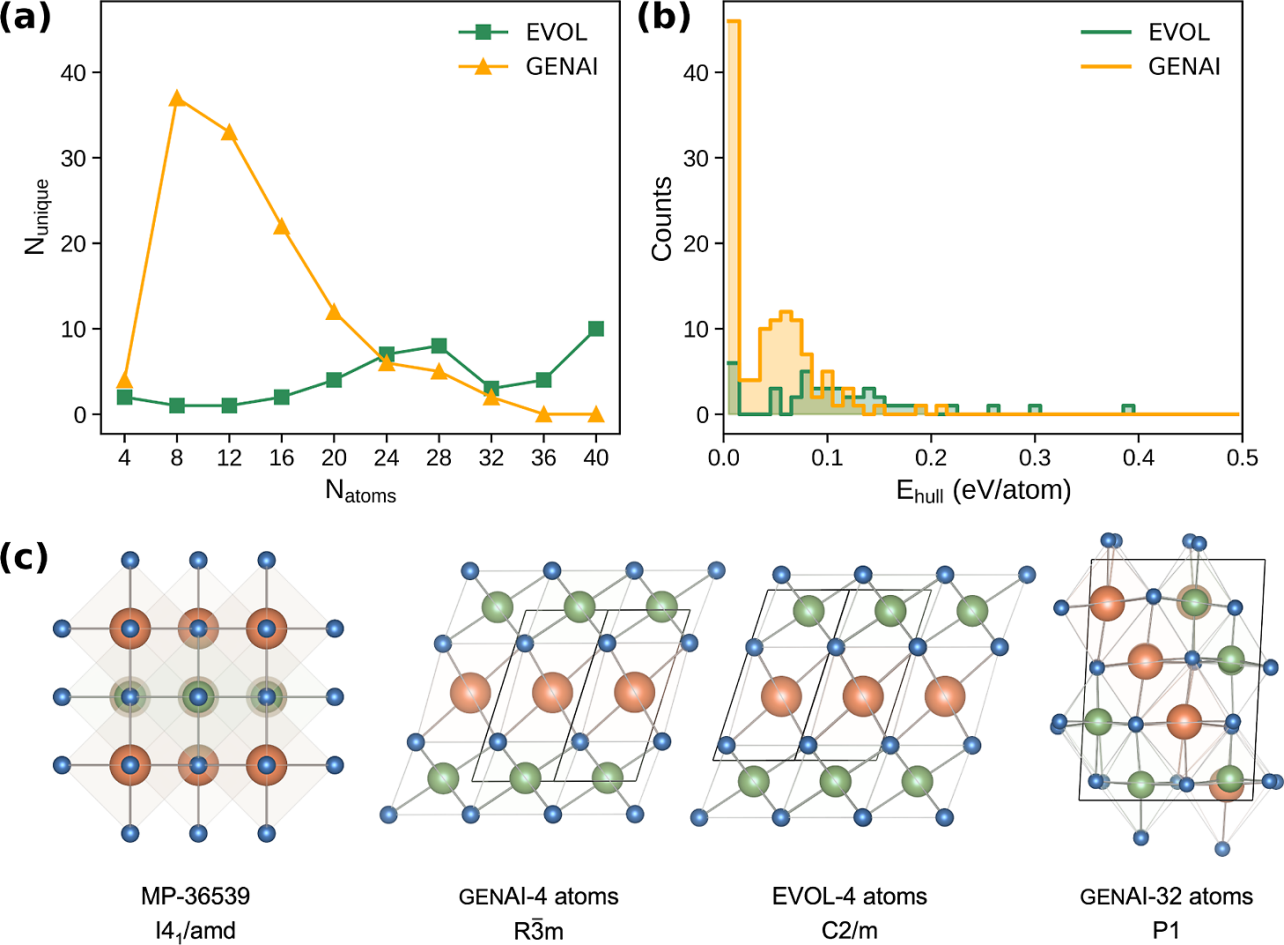
Comparison of structure generation between genAI and evol for KBiSe_2_. (a) Number of unique structures generated as a function of unit cell size (N_atoms_). (b) Distribution of predicted *E*_hull_. (c) Four representative stable structures. Atom colors: orange = K, green = Bi, blue = Se.

However, as the number of atoms increases, differences become more pronounced. genAI continues to produce a high number of chemically diverse and unique configurations, with a peak around 8 atoms per unit cell and strong representation in the 8–20 atom range. In contrast, evol explores cell sizes more uniformly but produces fewer structures in the small-cell regime. At larger cell sizes (>30 atoms), evol often generates geometrically irregular or chemically implausible configurations (as shown in Figure S7c), characterized by inconsistent coordination environments and deviations from typical octahedral frameworks in the K–Bi–Se chemical space. A limitation of the Chemeleon model used in this work is the conditioned generation based on text input. As the number of atoms increases, the model occasionally generates structures with compositions that deviate from the intended input. Since such mismatched structures are excluded from the final set, the overall number of unique structures decreases with increasing atom count. This issue could be alleviated by adopting models explicitly trained for crystal structure prediction, which solely learn atomic arrangements and lattice geometries, rather than relying on a text-conditioned generation framework.

These findings highlight a trade-off between the two approaches. While evol method offers broad and unconstrained exploration, they are susceptible to venturing into less chemically realistic regions of configuration space without strong priors. In contrast, the generative model benefits from learned structural biases, enabling it to efficiently sample chemically meaningful and synthetically plausible configurations even as structural complexity increases. We also compared the thermodynamic stability distributions of the structures generated by genAI and evol, as shown in Figure 6b. The results show that genAI samples a broader distribution of structures that are densely concentrated near the convex hull, indicating a stronger tendency to produce low-energy, and thus potentially synthesizable, configurations. While evol also identifies several low-energy structures, its overall distribution is more dispersed, with a larger fraction of structures exhibiting higher *E*_hull_. We do note that other implementations of evolutionary algorithms and related global optimization methods exist, and that these conclusions are based on a single model and comparison.

## Conclusion

Our comparative analysis highlights the strengths and limitations of distinct data-driven strategies for inorganic crystal structure prediction. While prototype substitution and evolutionary search continue to play valuable roles in exploring chemically plausible configurations, generative models such as Chemeleon demonstrate a marked advantage in producing diverse and thermodynamically favorable structures. In terms of computational cost, prototype substitution has the lowest overhead running in seconds on a modern workstation. Inference from the generative AI models is also fast, sampling hundreds of structures in minutes on a single GPU node. By leveraging uncertainty-aware machine learning force fields, we ensure robust ranking and improved reliability in candidate selection. The ability of generative AI to navigate complex energy landscapes and concentrate sampling near the convex hull underscores its potential as a transformative tool in materials exploration and discovery pipelines. Looking ahead, as such models mature, they will open the door to closed-loop discovery frameworks that couple generation, evaluation, and synthesis planning at scale.

## Supplementary Material



## Notes

DFT computational details for geometry optimization and thermodynamic stability calculations of generated structures. Figure S1: comparison of different methods with DFT in geometry relaxation and energy prediction. Figure S2: *t*-SNE embedding of SOAP descriptors with MP-ID labels. Figure S3: *t*-SNE embedding of SOAP descriptors with corresponding composition highlighted in orange color. Figure S4: distribution of predicted *E*_hull_ values for structures generated by SUB and genAI approach across 30 compositions. Figure S5: relation between the number of selected prototypes and the number of compositions with identified stable phases. Figure S6: Ca–Sr–S phase diagram with MP entries. Figure S7: representative structures generated by genAI and evol. All generative structures with predicted *E*_hull_ = 0 eV/atom are deposited into Zenodo with the link of 10.5281/zenodo.17493022, along with a JSON file that lists the CIF filenames and their corresponding MP-IDs, determined using StructureMatcher function based on the structural similarity (PDF)
