# Supplementary material for: Data-Driven Crystal Structure Prediction for Ternary Metal Chalcogenides
Source: Chem Mater. 2025 Dec 22;38(1):161–70. doi: 10.1021/acs.chemmater.5c02077 (PMC12805514; doi:10.1021/acs.chemmater.5c02077)
Supplement: Supplementary file 1 [file cm5c02077_si_001.pdf]

Supporting Information for: "Data-Driven Crystal Structure Prediction for Ternary Metal Chalcogenides"

Tianshu Li, Hyunsoo Park, and Aron Walsh*

Department of Materials, Imperial College London, Exhibition Road, London, SW7 2AZ, UK

E-mail: a.walsh@imperial.ac.uk

First-Principles Calculations

All first-principles calculations for evaluating were performed within the framework of density functional theory as implemented in Vienna *Ab initio* Simulation Package (VASP) using Pymatgen software.[1–4] The exchange-correlation energy was treated using the Perdew-Burke-Ernzerhof functional under the generalized gradient approximation.[5,6] The projector augmented-wave method described the interaction between valence electrons and ionic cores.[7,8] To keep consistent with the Materials Project (MP) to evaluate the thermodynamic stability of the generated structures, the geometry relaxation and the self-consistent-field calculations employed `MPRelaxSet` and `MPStaticSet` classes for each structure, respectively.

Phonon calculations were carried out using the finite-displacement method implemented in the PHONOPY package,[9,10] interfaced with VASP for force evaluations. Conventional unit cells of $SrCaS_2$ and Sr_3CaS_4 contain 4 and 8 atoms, and supercells of size $3\times3\times3$ and $3\times3\times1$ were constructed to ensure a sufficient interaction range for interatomic force constants.

Supporting Figures

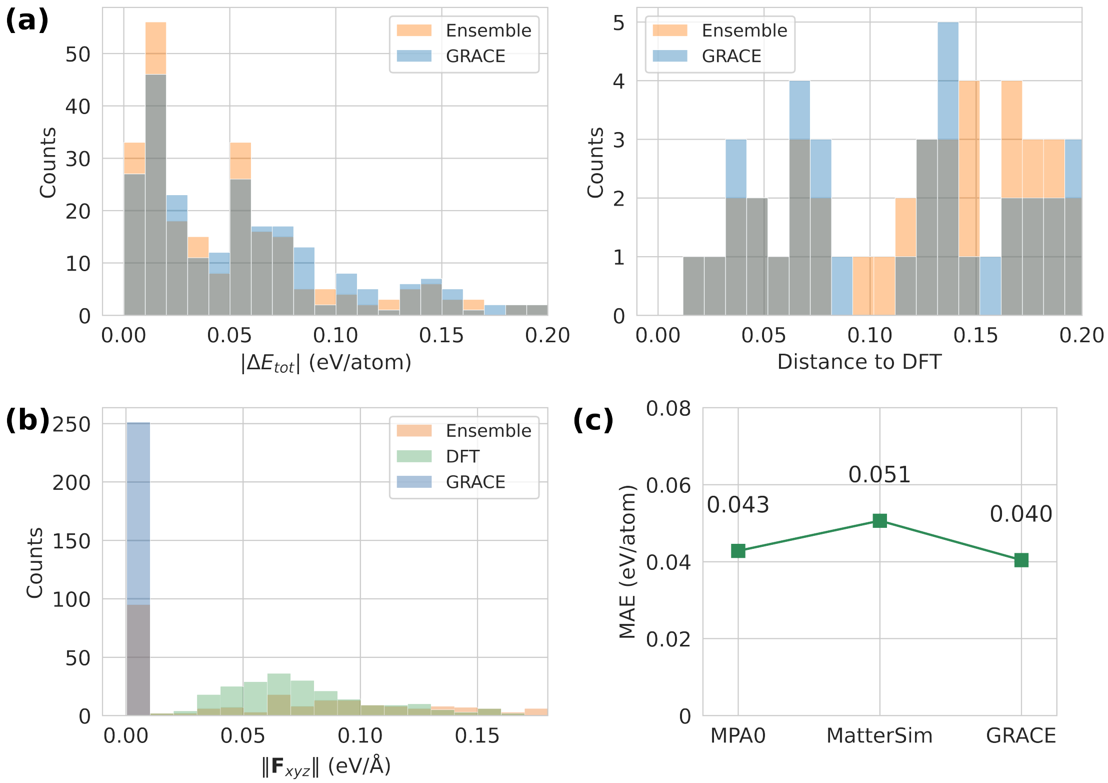

;

Figure S1: (a) Distribution of total energy differences and structural deviations between the structures optimized by ensemble MLIPs and `GRACE`, compared to DFT results. (b) Distribution of the mean norm of the atomic forces across x, y, and z directions by ensemble MLIPs (green), DFT (orange), and `GRACE` (blue). (c) The comparison of mean absolute error across three MLIPs.

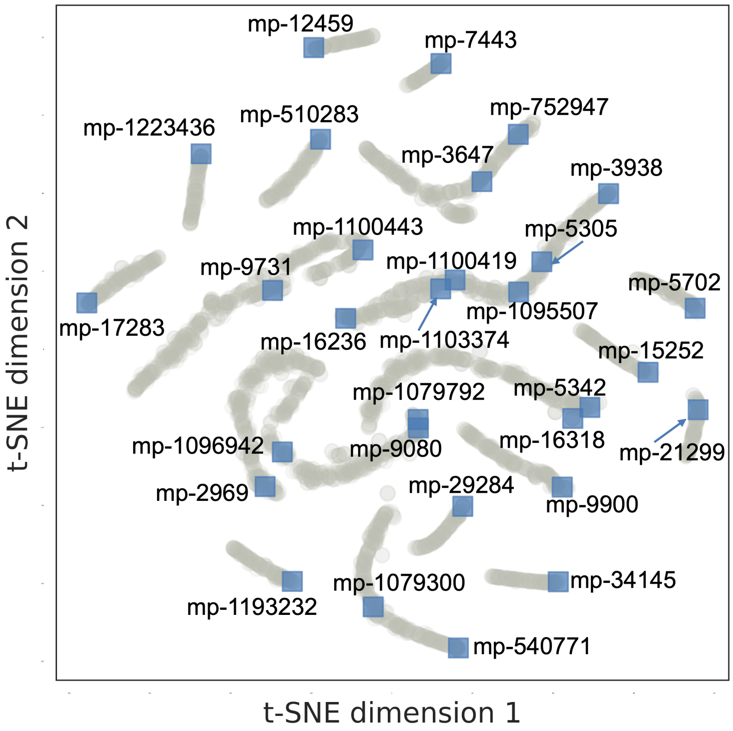

Figure S2: t-SNE embedding of SOAP descriptors illustrating the structural distribution of GENAI (grey circles and MP-reported lowest-energy structures (blue squares). Each square is labeled with the corresponding material ID.

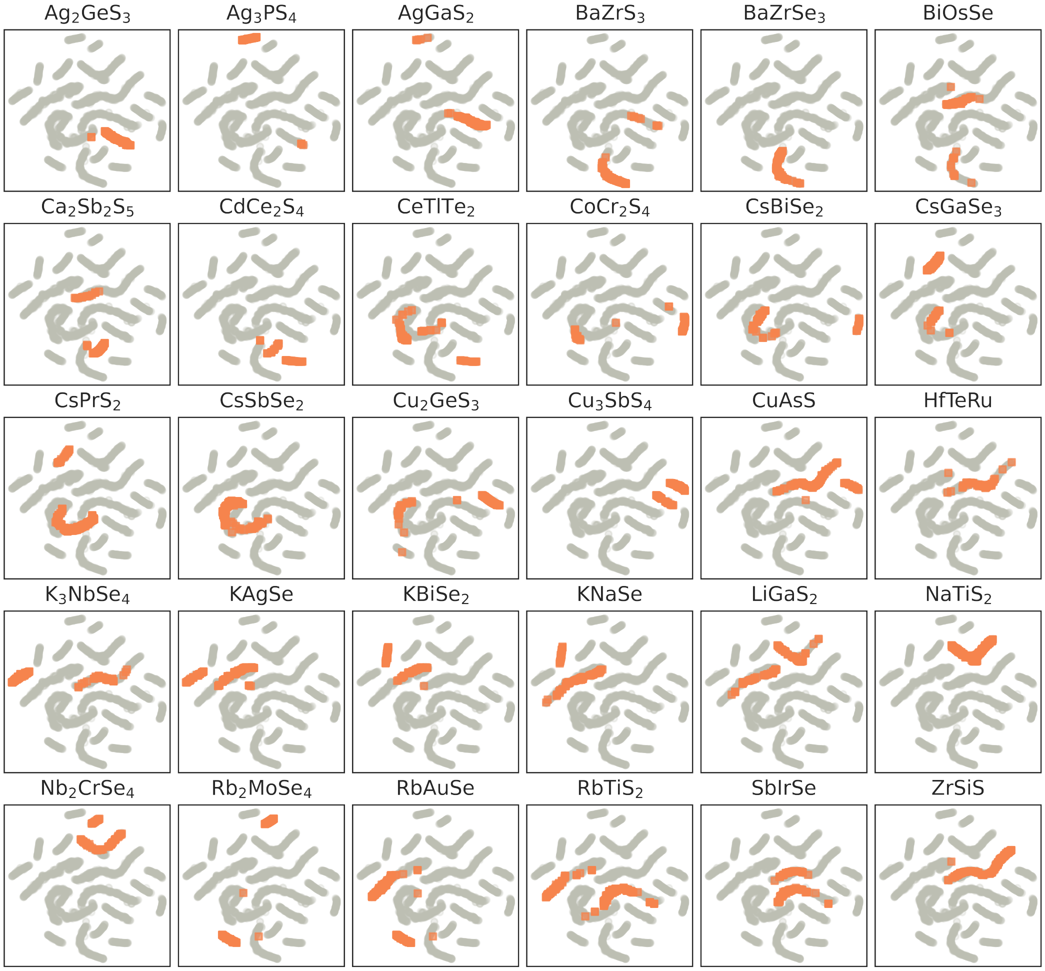

Figure S3: t-SNE embedding of SOAP descriptors showing the global structural distribution, with structures of a selected composition highlighted in orange color.

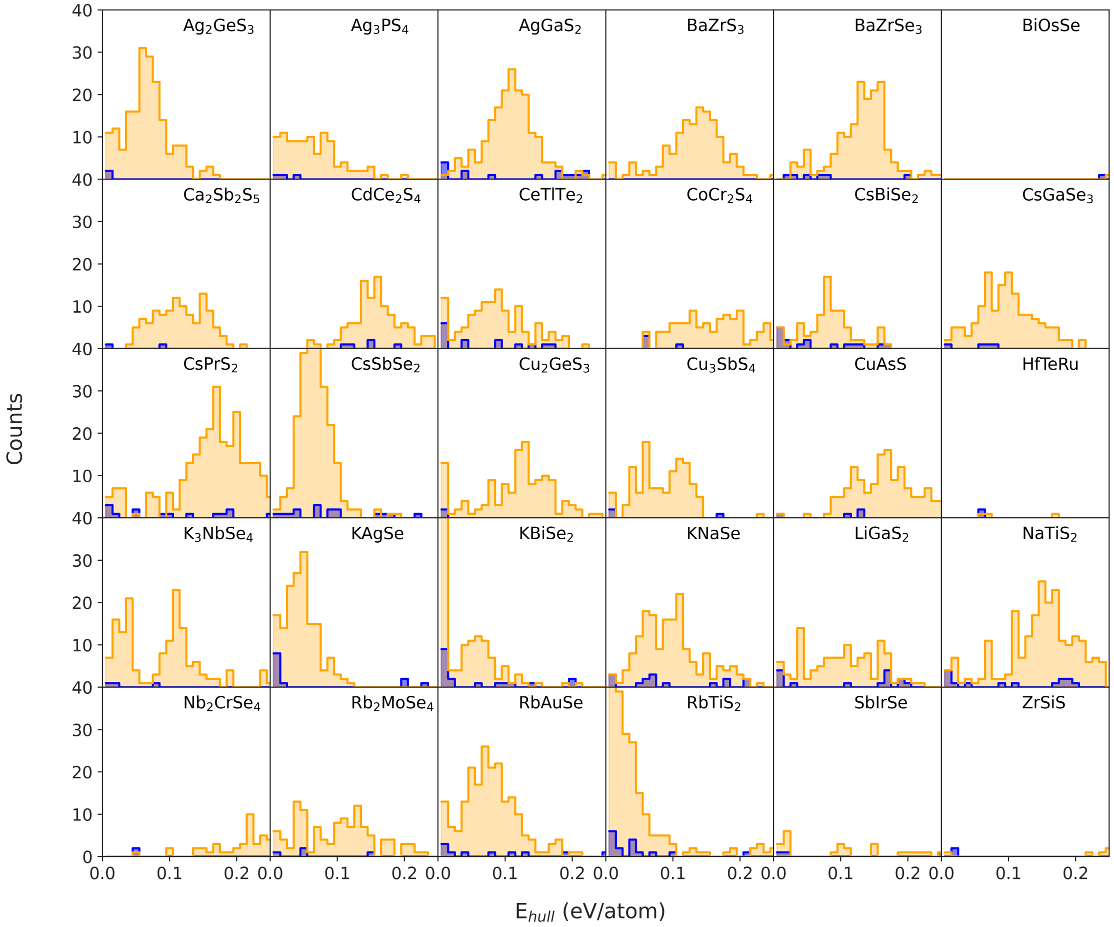

Figure S4: Distribution of E_{hull} predicted using MLIPs for structures obtained by SUB approach (blue) and GENAI (orange) approach across 30 compositions.

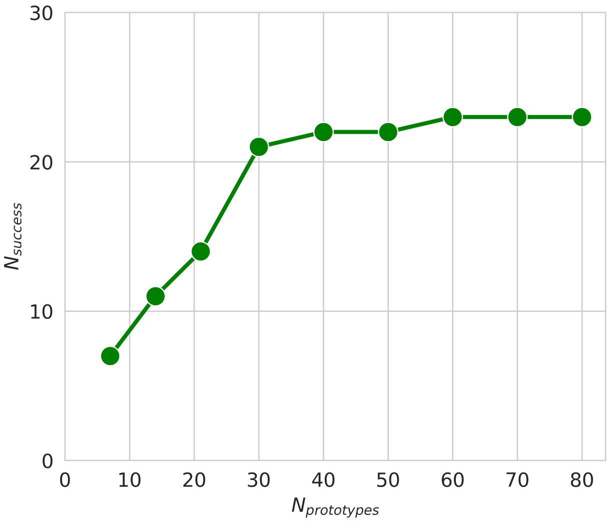

Figure S5: Relation between the number of selected structural prototypes (x-axis) and the number of compositions for which stable phases were identified (y-axis).

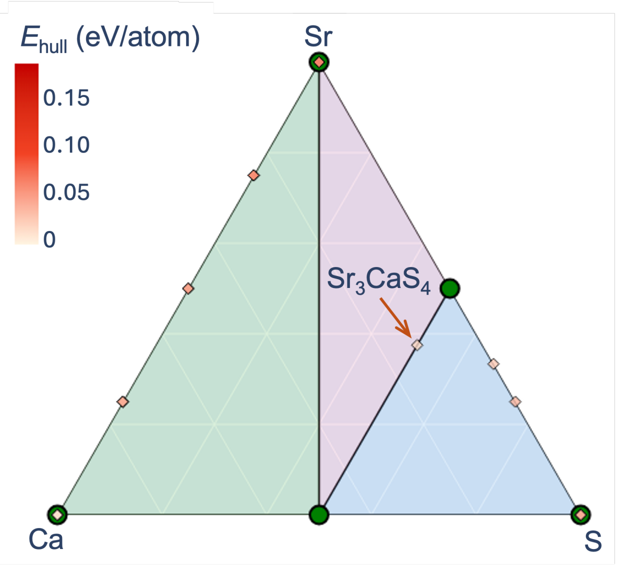

Figure S6: Ternary phase diagrams of the Ca–Sr–S system constructed using MP entries only. Green circles indicate thermodynamically stable phases, while red diamonds represent metastable structures. Color shading reflects the E_{hull} in eV/atom, with darker red indicating higher metastability.

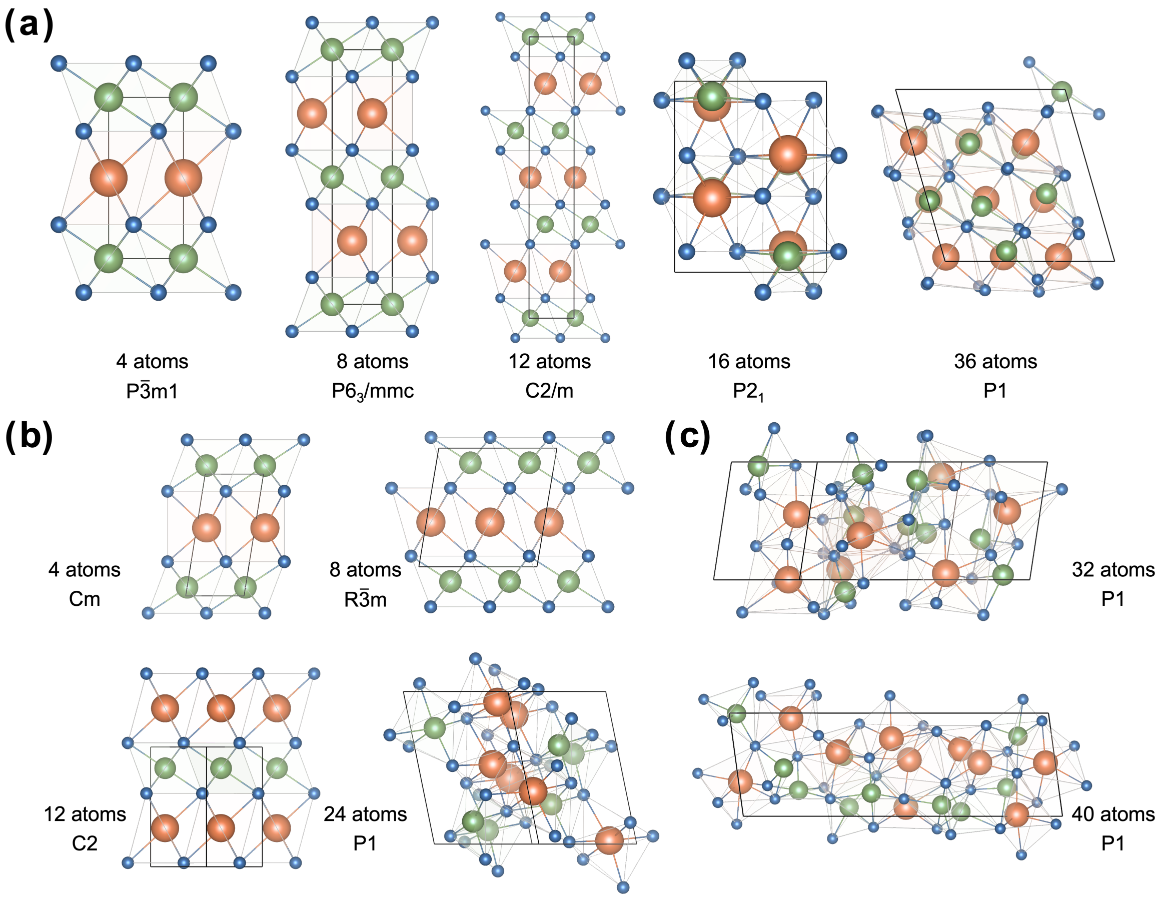

Figure S7: (a) Five predicted stable phases generated by `MAGUS`. (b) Four representative stable phases generated by `Chemeleon`. (c) Two representative phases with high factor generated by `MAGUS`. Atom colors: orange = K, green = Bi, blue = Se.

References

(1) Furthmüller, J.; Hafner, J.; Kresse, G. Dimer reconstruction and electronic surface states on clean and hydrogenated diamond (100) surfaces. *Phys. Rev. B* **1996**, *53*, 7334.

(2) Kresse, G.; Furthmüller, J. Efficiency of ab-initio total energy calculations for metals and semiconductors using a plane-wave basis set. *Comput. Mater. Sci.* **1996**, *6*, 15–50.

(3) Kresse, G.; Furthmüller, J. Software VASP, vienna (1999). *Phys. Rev. B* **1996**, *54*, 169.

(4) Jain, A.; Hautier, G.; Moore, C. J.; Ong, S. P.; Fischer, C. C.; Mueller, T.; Persson, K. A.; Ceder, G. A high-throughput infrastructure for density functional theory calculations. *Comput. Mater. Sci.* **2011**, *50*, 2295–2310.

(5) Ernzerhof, M.; Scuseria, G. E. Assessment of the Perdew–Burke–Ernzerhof exchange-correlation functional. *J. Chem. Phys.* **1999**, *110*, 5029–5036.

(6) Hammer, B.; Hansen, L. B.; Nørskov, J. K. Improved adsorption energetics within density-functional theory using revised Perdew-Burke-Ernzerhof functionals. *Phys. Rev. B* **1999**, *59*, 7413.

(7) Kresse, G.; Joubert, D. From ultrasoft pseudopotentials to the projector augmented-wave method. *Phys. Rev. B* **1999**, *59*, 1758.

(8) Blöchl, P. E. Projector augmented-wave method. *Phys. Rev. B* **1994**, *50*, 17953.

(9) Togo, A.; Chaput, L.; Tadano, T.; Tanaka, I. Implementation strategies in phonopy and phono3py. *J. Phys. Condens. Matter* **2023**, *35*, 353001.

(10) Togo, A. First-principles Phonon Calculations with Phonopy and Phono3py. *J. Phys. Soc. Jpn.* **2023**, *92*, 012001.
